# Long-Term Observation of the Quasi-3-Hour Large-Scale Traveling Ionospheric Disturbances by the Oblique-Incidence Ionosonde Network in North China

**DOI:** 10.3390/s22010233

**Published:** 2021-12-29

**Authors:** Ruijiao Zhang, Gang Chen, Yaxian Li, Shaodong Zhang, Wanlin Gong, Zhiqiu He, Min Zhang

**Affiliations:** Electronic Information School, Wuhan University, Wuhan 430072, China; zhangruijiao@whu.edu.cn (R.Z.); yaxianli@whu.edu.cn (Y.L.); zsd@whu.edu.cn (S.Z.); gongwl@whu.edu.cn (W.G.); zqhe1234@whu.edu.cn (Z.H.); m1nzhang@whu.edu.cn (M.Z.)

**Keywords:** ionospheric remote sensing, large-scale traveling ionospheric disturbances, oblique-incidence ionosonde, wind field

## Abstract

The oblique-incidence ionosonde network in North China is a very unique system for regional ionospheric observation. It contains 5 transmitters and 20 receivers, and it has 99 ionospheric observation points between 22.40° N and 33.19° N geomagnetic latitudes. The data of the ionosonde network were used to investigate the statistical characteristics of the quasi-3-h large-scale traveling ionospheric disturbances (LSTIDs). From September 2009 to August 2011, 157 cases of the quiet-time LSTIDs were recorded; 110 cases traveled southward, 46 cases traveled southwestward and only 1 case traveled southeastward. The LSTIDs mainly appeared between 10:00 and 19:00 LT in the months from September to the following May. We compared the data of the Beijing, Mohe and Yakutsk digisondes and found that the LSTIDs are most likely to come from the northern auroral region. These LSTIDs may be induced by the atmospheric gravity waves (AGWs) and presented obvious seasonal and diurnal varying features, indicating that the thermospheric wind field has played an important role.

## 1. Introduction

Large-scale traveling ionospheric disturbances (LSTIDs) are generally considered as the manifestation of the atmospheric gravity waves (AGWs) in the ionosphere and are usually observed while the geomagnetic storms and auroral substorms occur [1,2,3,4,5]. Joule heating and Lorentz forces caused by the enhancement of the auroral electrojet or intense precipitation of charged particles are the main excitation sources of the LSTIDs, and they have typical horizontal velocities between 300 and 1000 m/s, horizontal wavelengths of more than 1000 km and period in the range of 30 min to 5 h. These periodic waves carry huge energy from the lower neutral atmosphere to the upper ionosphere and induce periodic electron density fluctuations in the ionosphere [4,6]. Almost all of the reported individual LSTID events occur during geomagnetic storms or auroral substorms (e.g., [7,8,9,10]). There are also a number of statistical analyses of the LSTIDs. The LSTIDs were found to occur in both the magnetically quiet and disturbed days according to the total electron content (TEC) data of the GPS observation network in Japan [11]. The statistical study of the TEC data in Europe shows that the amplitude of the LSTIDs has an obvious positive correlation with the AE index [12]. The occurrence rate of the LSTIDs in North America presents peaks at 12:00 and 19:00 LT [13]. The occurrence rate of the LSTIDs in China displays a maximum in winter and a minimum in summer [5]. In brief, there are many reports about the LSTIDs, and they come to different conclusions. However, very few reports specifically targeted the magnetically quiet LSTIDs.

A variety of equipment types, such as ionosondes [14,15,16], incoherent scatter radars [17,18,19,20], satellite beacons [21], HF Doppler radars [22] and airglow imagers [23,24,25], can be used to observe LSTIDs. LSTIDs span thousands of kilometers with very long wavelength. One piece of radio equipment can only observe one spot of the ionosphere. The airglow imager can observe a large area of the ionosphere but only operates at night. Nowadays, the high-density observations of large area ionosphere are mainly performed by the GPS receiver network. The TEC recorded by a GPS receiver is the total number of the electrons along the satellite–ground link. A vertical-incidence (VI) ionosonde operating in the high-frequency (HF) band can measure more parameters of the ionosphere, such as the maximum electron density and peak height of each ionospheric layer. Theoretically, the peak electron density variation gained through the swept-frequency ionogram can accurately reflect many kinds of ionospheric disturbance. Considering the cost of construction, it is impossible to set up a dense detection network composed of the VI ionosondes. However, the oblique-incidence (OI) ionosonde is a kind of low-cost system for network observation. We used the data of the OI ionosonde network in North China to study the mid-latitude LSTIDs. In Section 2, the detection network and the data analysis method are introduced. The typical examples of the LSTIDs and the statistical characteristics for two-year observation are presented in Section 3. In Section 4, the possible source and the reason for the diurnal and seasonal dependence of the recorded LSTIDs are discussed. A conclusion is made in the last section.

## 2. Typical Observation Cases

**Figure 1:**
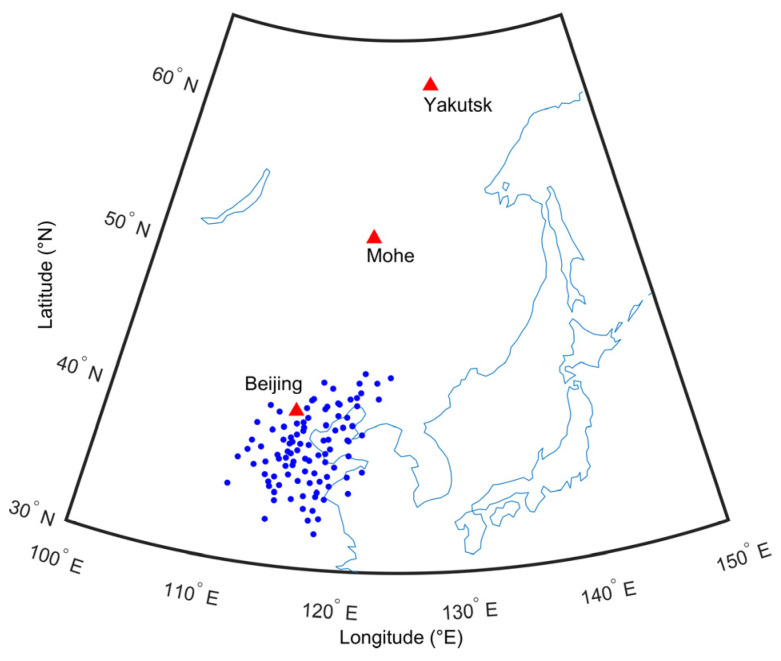
Geographical locations of the observation points in Eastern Asia. The 99 blue circles indicate the ionospheric observation points of the oblique-incidence ionosonde network. The three red triangles show the locations of the Beijing, Mohe and Yakutsk digisondes.

As shown in Figure 1, the OI ionosonde network in North China and three digisondes were used to investigate the large-scale oscillations in the ionosphere. The three digisondes are located in Beijing (40.3° N, 116.2° E), Mohe (53.5° N, 122.37° E) and Yakutsk (62.0° N, 129.6° E). Their geomagnetic latitudes are 30.2° N, 43.4° N, and 52.4° N, respectively, and are indicated as red triangles in Figure 1. They record ionograms from 0.5 to 12 MHz with 50 kHz step every 15 min. The ionospheric parameters as well as the electron density profile of the ionospheric F2 layer can be derived from the manually scaled trace of the ordinary waves by the SAO-Explorer [26]. The blue dots display the ionospheric observation points of the OI ionosonde network in North China.

**Figure 2:**
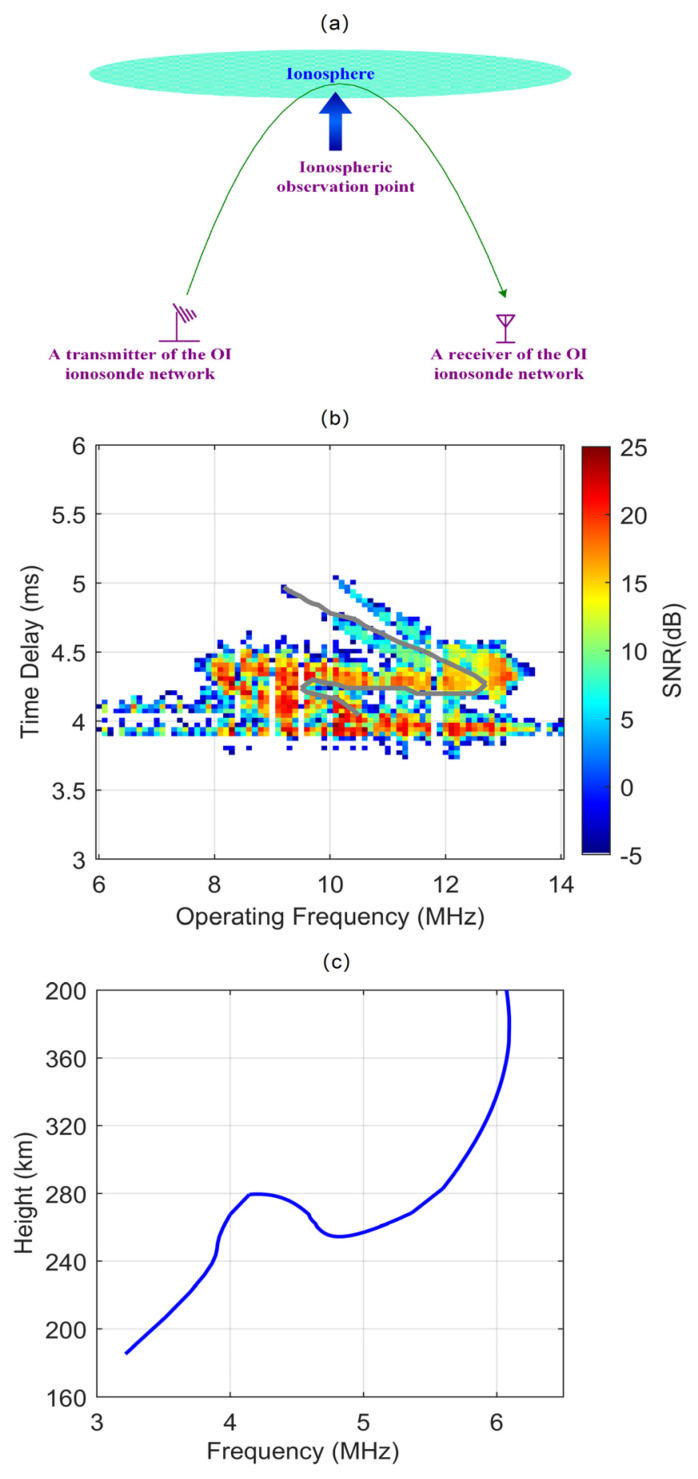
(**a**) Schematic diagram for ionospheric oblique-incidence detection. (**b**) A typical oblique-incidence ionogram with ordinary and extraordinary waves. The gray curve is the echo trace of the F1 and F2 layer ordinary wave. (**c**) The converted vertical-incidence echo trace through the gray curve in plot (**b**).

Different from the traditional VI ionosonde, the transmitter and receiver of an OI ionosonde are separated, and it can also measure the ionospheric parameters such as the virtual and peak heights, as well as the critical frequency, like a traditional VI ionosonde [27,28]. As shown in Figure 2a, the ionospheric observation point is in the middle between the transmitter and receiver of an OI ionosonde. The OI ionosonde also operates in swept-frequency mode and records the echoes of each operating frequency. A typical swept-frequency OI ionogram is displayed in Figure 2b, and the echo trace of the ordinary waves is manually scaled and indicated by the gray curve. The distance between the transmitter and receiver is 1152.78 km. There is a certain relationship between the oblique-incidence ionogram and the vertical-incidence ionogram. The oblique echo trace is converted to the vertical ordinary trace by Martyn’s equivalent path theorem with the assumption of a spherically stratified ionosphere and no magnetic field [29]. The converted echo trace is shown in Figure 2c, and the blue curve displays a clear vertical echo trace of ionospheric F1 and F2 layers. The critical frequency of the F2 layer (*f_o_*F2) used in the following is obtained by this echo trace conversion method, and the long period observation verified the accuracy of this method with the comparison of the VI observation [30,31]. Chen et al. [31] made an experimental comparison between oblique and vertical detections. They believed that the deviations of *f_o_*F2 between the vertical and oblique observations never exceed 1 MHz and are generally less than 0.5 MHz.

In a network consisting of many OI ionosondes, the signal of one transmitter can be received by several receivers, and one receiver can receive the signal of several transmitters; thus, fewer radio systems can be used to obtain more ionospheric observation points. In North China, the OI ionosonde network composed of 5 transmitters and 20 receivers was constructed to observe the regional ionosphere with 99 observation points [32]. The blue circles in the map of Figure 2 display the observation points of the ionosonde network in North China. The observation area covers the latitudes between 32.34° N and 43.03° N, the longitudes between 111.35° E and 124.43° E and the geomagnetic latitudes between 22.40° N and 33.19° N. The ionosonde network records 99 OI ionograms every 30 min. Through the OI echo trace inversion method, we have estimated the *f_o_*F2 at all the observation points of the ionosonde network. In order to eliminate the day-to-day variations and so highlight the periodic disturbances in the F2 layer, we estimate the difference *f_o_*F2 (∆*f_o_*F2) compared with the monthly value,

∆*f_o_*F2 = (*f_o_*F2 − *f_o_*F2_m_)/*f_o_*F2_m_ (1)

where *f_o_*F2_m_ represents the monthly mean of the *f_o_*F2 during the magnetically quiet days.

The data of the OI ionosonde network from 1 September 2009 to 21 August 2011 are analyzed. Except for the maintenance time, the network has been working for 684 days. Excluding the impact of geomagnetic activities, there are 595 magnetically quiet days with the maximum Kp index ≤ 3. During these days, 157 cases of the LSTIDs from the north have been recorded. The observed LSTIDs mainly traveled to the south and southwest. We introduce two typical cases of them firstly.

**Figure 3:**
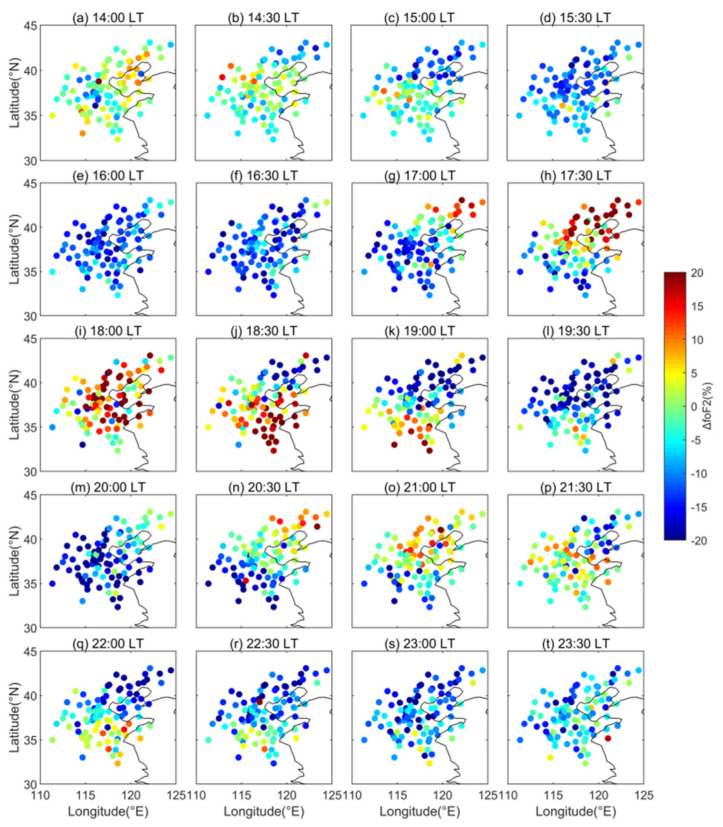
Maps showing the Δ*f_o_*F2 variations at all the observation points of the ionosonde network from 14:00 to 23:30 LT with half an hour step on 24 November 2010.

**Figure 4:**
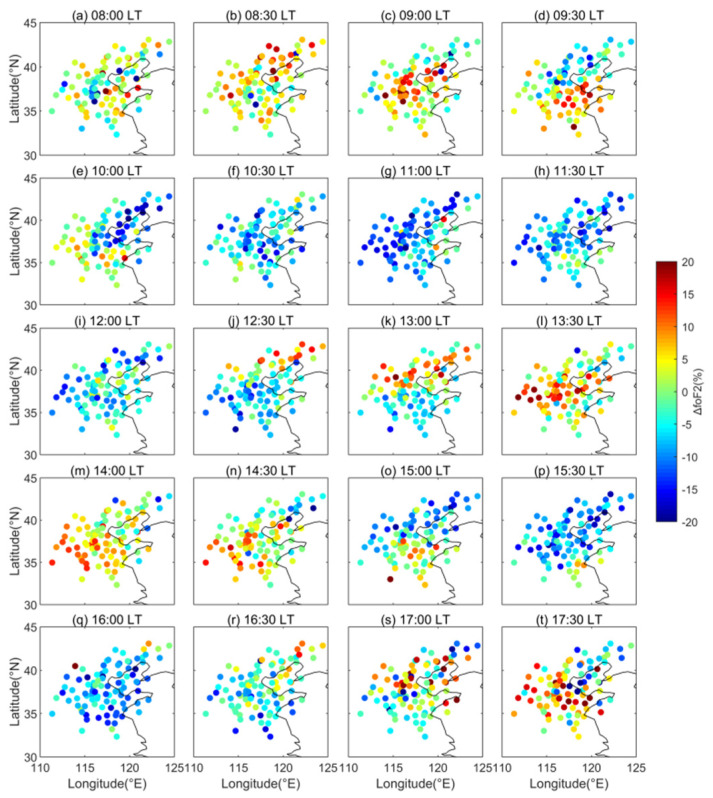
Maps showing the Δ*f_o_*F2 variations at all the observation points of the ionosonde network from 8:00 to 17:30 LT with half an hour step on 22 January 2011.

One case of the LSTID occurring on 24 November 2010 is shown in Figure 3. The *f_o_*F2 depletion firstly appeared at 14:30 LT in the top right corner of Figure 3b and then extended southwestward to cover all the observation area at 16:30 LT. At 17:00 LT, the *f_o_*F2 enhancement emerged in the top right corner of Figure 3g and also moved to the southwest. At 20:00 LT in Figure 3m, the regional enhancement had moved out of the observation region. Soon after, the second circle of the *f_o_*F2 enhancement occurred in the northeast in Figure 3n. The regional enhancement traveled southwest through the observation region and moved out at 23:00 LT. The other typical case was recorded on 22 January 2011 and is displayed in Figure 4. The regional *f_o_*F2 enhancement emerged in the north of the map in Figure 4b at 8:30 LT and then moved southward. At 10:30 LT in Figure 4f, it propagated out of the observation area. The second and third circles of the southward moving *f_o_*F2 enhancement appeared at 12:30 LT in Figure 4j and at 16:00 LT in Figure 4q, respectively. Both cases present the obvious periodic oscillations traveling across the North China ionosphere.

**Figure 5:**
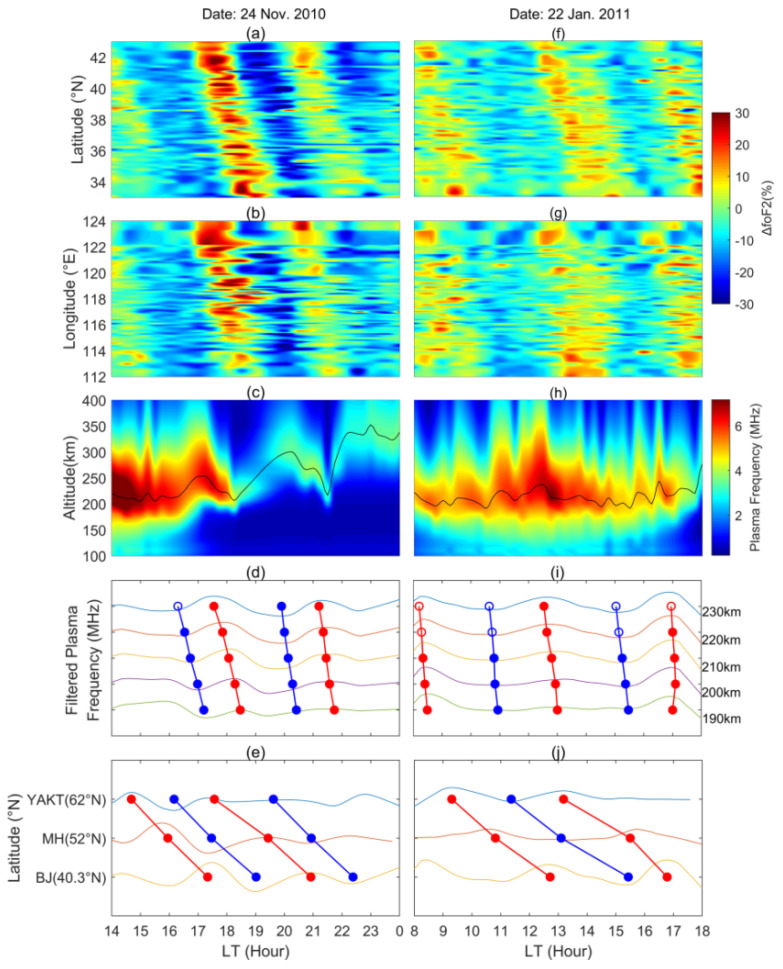
Two typical examples of the LSTIDs that occurred between 14:00 and 24:00 on 24 November 2010 (**a**–**e**) and between 8:00 and 18:00 on 22 January 2011 (**f**–**j**). The first row shows the latitude dependence of the LSTIDs. The second row shows the longitude dependence of the LSTIDs. The third row shows the ionospheric electron density profiles recorded by the Beijing digisonde, and the black curves indicate the peak height of the F2 layer (*h_m_*F2). The fourth row shows the filtered plasma frequency variations at five selected heights. The red and blue circles represent the peak and trough of the observed fluctuations, and the solid lines connect the circles at different heights. The hollow circles indicate the data above the peak height of the F2 layer. The bottom row displays the filtered F2-layer critical frequency (*f_o_*F2) recorded by the Yakutsk (YAKT), Mohe (MH) and Beijing (BJ) digisondes. The red and blue circles represent the peak and trough on the *f_o_*F2 curves, and the solid lines connect the circles to show the wave propagating tendency.

The latitude and longitude dependences of the two cases of ionospheric oscillations are drawn in the top two rows of Figure 5. The oscillations recorded on 24 November 2010 and 22 January 2011 are displayed in the left and right columns of the figure, respectively. The first row shows the latitude–time dependence of the ∆*f_o_*F2 values recorded by the 99 observation points. The disturbances of several periods are clearly displayed. They lasted for more than 10 h with periods of 3.9 h in Figure 5a and 4.3 h in Figure 5f. The wave peaks and troughs slanting to the right in both plots indicate the southward moving tendency. The longitude–time dependence of the ∆*f_o_*F2 values is displayed in the second row of Figure 5, and the obvious fluctuation features are also present in these two plots. The peaks and troughs in Figure 5b move to the smaller longitudes, indicating the westward zonal velocity. In Figure 5g, the peaks and troughs at different longitudes appeared almost simultaneously, implying that the waves were propagating to almost due south. According to the slopes of wave peak and trough in the latitude–time and longitude–time plots, the horizontal speed and the azimuth of the oscillation in the left column are estimated as 169.03 ± 13.92 m/s and 221.57 ± 0.56°, and the same parameters in the right column are 218.24 ± 12.88 m/s and 182.18 ± 0.31°. The computing method for the wave speed and azimuth is provided in the Appendix A. This algorithm can accurately calculate the parameters we need.

To further study the characteristics of the two LSTIDs, the observations of the three digisondes at different latitudes are analyzed. The third row of Figure 5 displays the electron density profiles estimated from the ionograms of the Beijing digisonde. The black curves display the variations of the peak height of the F2 layer (*h_m_*F2). The variations of the plasma frequency at the heights from 190 to 230 km with 10 km step are presented in the fourth row of Figure 5. The displayed plasma frequency curves have passed through the band-pass filter of 1–6 h. The periodic fluctuations appear on all the displayed heights and they are consistent with the wave phase in the top two rows. The solid and hollow circles indicate the data below and above the *h_m_*F2, respectively. We connect the wave peaks and troughs at different heights and find that the wave phase velocities are downward. The echoes of an ionosonde are reflected below *h_m_*F2, so the solid circles in Figure 5d,i are applied to estimate the vertical phase velocities. According to the slope of the connecting lines, the vertical phase velocities of the two LSTIDs are estimated as 16.81 ± 4.69 and 32.74 ± 8.93 m/s downward, respectively. Usually, most of the large-scale disturbances in the ionosphere stem from the lower atmosphere, and the downward phase velocities indicate that they are very likely the upward gravity waves [33,34,35]. The filtered *f_o_*F2 curves recorded by the digisondes in Beijing, Mohe and Yakutsk are shown in the bottom row of Figure 5. The band-pass filter of 1–6 h is also applied. The wave peaks and troughs are connected by the slope lines, which indicate the southward propagating velocities of the LSTIDs. Due to the very long distance between the three digisondes, the traveling speed of the waves cannot be accurately estimated, but it clearly illuminates that the recorded LSTIDs come from the auroral region north of Yakutsk.

## 3. Statistical Analysis

**Figure 6:**
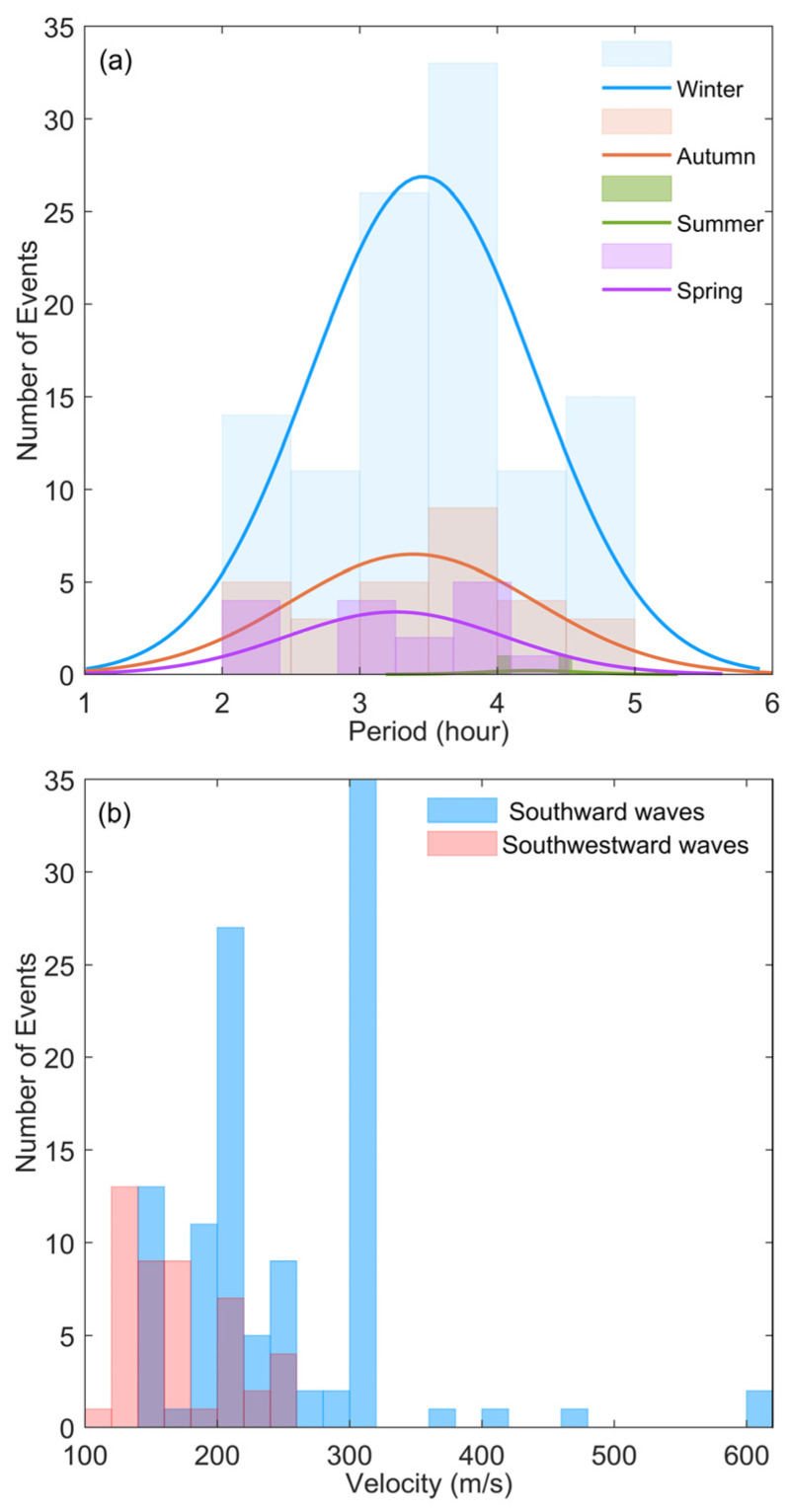
(**a**) Period distribution of the LSTIDs from the north in different seasons between 1 September 2009 and 21 August 2011. The solid curves are the Gaussian fitting of the histograms. (**b**) Horizontal velocity distribution of the LSTIDs propagating southward and southwestward.

We develop four criteria to accurately recognize the LSTIDs from other ionospheric disturbances: (1) The amplitude of the enhancement or depletion exceeds the diurnal average by at least 5%. (2) At least 60% of the ionospheric observation points of the ionosonde network as well as the Beijing digisonde have recorded the same periodic disturbance. (3) The duration of the wave peak and trough exceeds 30 min. (4) More than two circles of the disturbances are recorded. In the magnetically quiet days from 1 September 2009 to 21 August 2011, 157 cases of the LSTIDs from the north have been recorded by the OI ionosonde network. The total occurrence rate of the quiet-time LSTIDs is 26.39%, which is defined as a fraction of the days the quiet-time LSTIDs were observed against the total number of magnetically quiet days. The four seasons in North China are defined as spring (March–April), summer (May–August), autumn (September–October), and Winter (November–February). The occurrence number and occurrence rate of the quiet-time LSTIDs in four seasons are presented in Table 1. The LSTIDs mainly occur in winter and can be observed almost every two days. In summer, only two cases have been recorded. The occurrence rate in autumn is 10.7% more than that in spring. This is consistent with the previous observations of Tang et al. [36]. The periods of the LSTIDs recorded in the four seasons all tend to have Gaussian distribution between 2–5 h, as shown in Figure 6a, and they are centered at 3.44 h. Figure 6b shows the distribution of the horizontal velocities of the southward and southwestward traveling disturbances, respectively. The velocities of the southwestward waves vary mainly between 106.67 and 256.01 m/s, and those of the southward waves are mainly between 153.12 and 312.52 m/s. The median azimuths and the standard deviations of the southward and southwestward traveling LSTIDs are 181.58 ± 14.43° and 224.98 ± 17.43°, respectively.

**Figure 7:**
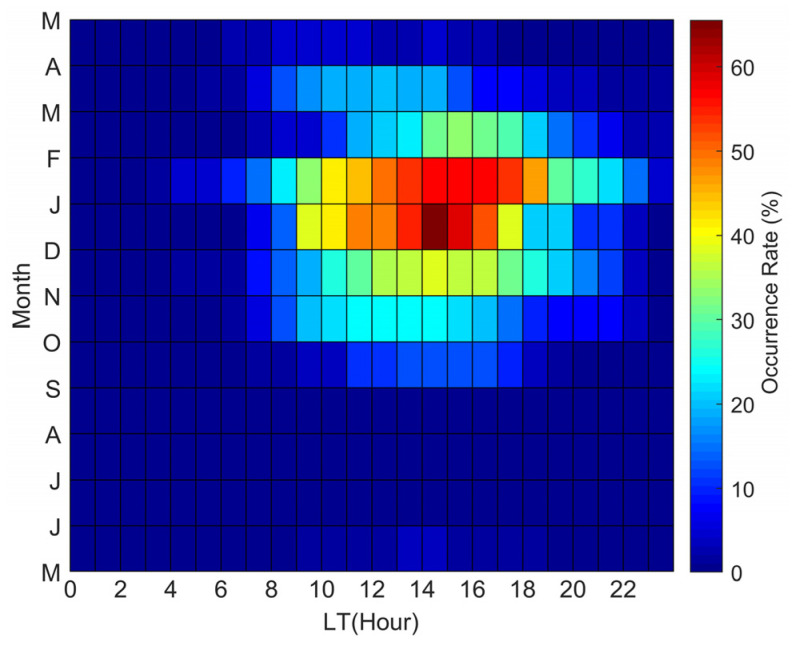
Time–month map of the occurrence rate of the LSTIDs. LT = UT + 8 h.

The occurrence rates of the LSTIDs varying with local time in different months are shown in Figure 7 and present obvious diurnal and seasonal dependence. The occurrence rate is defined as a fraction of the number of days of the quiet-time LSTIDs observed against the total number of magnetically quiet days of a given month, the same method used by Otsuka et al. [37]. The mid-latitude LSTIDs appear mainly between 9:00 and 19:00 LT from September to March. The statistical study of Tsugawa et al. [11] also showed that under the quiet conditions of Kp ≤ 3, the occurrence number of the LSTIDs was much larger in the daytime than in the nighttime.

**Figure 8:**
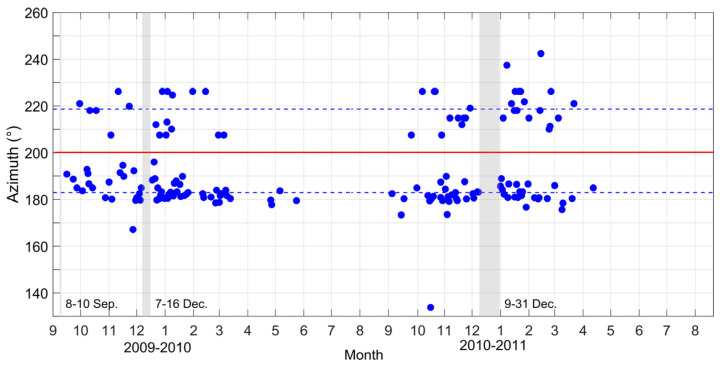
Azimuth variation of the recorded LSTIDs between September 2009 and August 2011. The azimuths are measured clockwise from north. The red horizontal line shows the boundary between the LSTIDs propagating southwestward and southward. The two dotted blue horizontal lines show the mean azimuths of the southwestward and southward traveling LSTIDs, respectively. The vertical gray bars are used to indicate the unoperated period of the ionosonde network.

Two-year azimuth variations of the mid-latitude LSTIDs are presented as the blue dots in Figure 8. Except for a few days of downtime as indicated by the gray vertical bars, the ionosonde network was well operated from 1 September 2009 to 21 August 2011. As shown in Figure 8, the recorded LSTIDs come from about three fixed directions. Only one southeastward LSTID emerged on 17 October 2010. There is an obvious boundary between the southwestward and southward traveling LSTIDs as shown by the red horizontal line at 200° azimuth. There are 46 cases above the red horizontal line traveling southwestward with the azimuth between 207.55 and 242.40°. Their average azimuth is 218.66° and is indicated by the dotted blue horizontal line above. There are 110 cases below the red horizontal line propagating southward with the azimuth between 167.14 and 196.01°. The dotted blue horizontal line below is used to indicate their average azimuth of 182.95°. It is very likely that the two kinds of LSTIDs stemmed from two different sources. In the studies of the ionospheric disturbances induced by earthquakes, thunderstorms and so on [35,38,39,40], the waves traveled from the source to all directions, and the measured wave direction depends on the orientation of the wave source relative to the observer. Both the southwestward and southward LSTIDs present the same seasonal characteristics. They appeared mainly from September to the following May and disappeared from June to August. It is very likely that the same background factor dominates the propagation of the LSTIDs in two different directions.

## 4. Discussion

Almost all the case studies of the LSTIDs focus on the disturbances associated with storms (e.g., [10,41,42,43]). Some statistical studies also considered that LSTIDs are strongly linked to the geomagnetic activity at high latitudes (e.g., [13,36,44]). However, there are a few investigations about the LSTIDs occurring during the magnetically quiet days, and much fewer investigations concern the LSTIDs with periods larger 2 h (e.g., [5,45]). We attempted to study the LSTIDs with periods larger than 1 h using the OI ionosonde network in North China. According to the two-year observations, we have found interesting LSTIDs with periods concentrated around 3.44 h. Most of the LSTIDs came from north and northeast, and their downward phase velocities between 190 and 230 km altitudes indicated that they may travel upward from the lower atmosphere and be related to AGWs. Compared with the observations of the three digisondes in different latitudes from high to mid-latitude, we find that both the southward and southwestward propagating LSTIDs stemmed from the north auroral region. However, these quiet-time LSTIDs have a larger period than the disturbed-time LSTIDs reported by Tsugawa et al. [11]. Quiet-time LSTIDs are often considered to be excited by the AGWs moving equatorward from a source in the dawn sector of the auroral zone [46]. The momentum and energy disturbed by the AWG are transmitted to the charged particles through the collision between neutral particles and charged particles. Thus, the periodic disturbances travel into the ionosphere from the neutral atmosphere to generate the TID along its propagation path.

Most AGWs are generated in the lower atmosphere and travel upward. Previous studies by many scholars have shown that the propagation of AGWs is very sensitive to the filtering effect of the background wind. Therefore, the propagation direction of gravity waves exhibits obvious seasonal characteristics. Medeiros et al. [47] investigated the dominant quasi-monochromatic gravity waves in the upper mesosphere from September 1998 to October 1999 using the all-sky imager at Cachoeira Paulista, Brazil (23° S, 45° W), and found the waves exhibiting an obvious seasonal dependence on the horizontal propagation direction, propagating toward the southeast during the summer months and toward the northwest during the winter, which were attributed to a strong filtering of the waves in the middle atmosphere by the stratospheric winds. Takeo et al. [48] analyzed the 557.7 nm airglow images obtained at Shigaraki MU Observatory (34.8° N, 136.1° E) and found the propagation direction of gravity waves presented clear seasonal variation in the mesopause region. The propagation direction of the gravity waves in the mesopause region was mainly controlled by the wind filtering. The global gravity wave variations in the thermosphere were investigated by Miyoshi et al. [49]. The gravity waves were found propagating upward from the lower atmosphere, and the wave energy at the thermospheric altitudes reveals obvious seasonal dependence, indicating the filtering effect of the background wind. The TIDs are suggested as the plasma density manifestations of the gravity waves at ionospheric F-layer heights [50]. When a gravity wave propagates upward in the atmosphere, its wave amplitude becomes larger and larger due to the lower and lower atmospheric density with the altitude increasing. Most of the upward gravity waves will break before they pass through the mesopause, and some of them can reach the heights of the thermosphere and modulate the plasma to form the periodic ionospheric disturbances. The downward phase velocities of the recorded LSTIDs indicate that they may be induced by the AGWs traveling upward. The statistics of the quiet-time LSTIDs from both north and northeast present similar seasonal dependence, namely the highest occurrence rate in winter and the lowest in summer, which implies the possible wind filtering effect in the atmosphere.

According to the law of energy conservation, the amplitude of a gravity wave increases as the atmospheric density decreases with height. When reaching the MLT region, the wave amplitude is large enough to generate instabilities, which would lead to wave breaking when the temperature lapse rate of the waves plus the mean flow becomes super-adiabatic [51,52,53,54,55]. Therefore, the background wind field at these heights and above becomes very important for the upward traveling waves. In the thermosphere, the neutral winds blow poleward during daytime and equatorward during nighttime due to the pressure gradients caused by the solar EUV heating of the thermosphere. Regarding the seasonal variation of the thermospheric winds, the wind blows from the summer hemisphere to the winter hemisphere. Consequently, the poleward wind during the daytime is stronger in winter than in summer. In Figure 7 and Figure 8, it can be seen that the diurnal and seasonal dependences of the LSTIDs are closely related to the thermospheric meridian wind field. The 2–5 h LSTIDs can be observed at mid-latitudes when they are traveling in the opposite direction of the mean meridian winds. When the mean meridian winds turn equatorward, both the southward and southwestward LSTIDs disappear at mid-latitudes. As is well known, the wind field in the same direction of the phase velocity of an AGW will stretch the AGW rays in the propagation direction and increase the energy attenuation of the waves in the atmosphere, thus hindering the AGW propagation. Conversely, when the AGW encounters a reverse wind field, the AGW rays will shrink to shorten the propagation path of the AGW, and thus the energy attenuation of the wave will be reduced to form a favorable environment for the wave propagation [56,57]. According to the dispersion relation for gravity waves, the vertical wavelength of a gravity wave becomes small when the background wind blows in the same direction as the gravity wave propagation, and thus the gravity waves tend to be dissipated due to high viscosity in the thermosphere. As a result, the gravity waves in winter can survive. This suggests that the atmospheric background wind field could play an important role in the LSTIDs’ appearance.

The seasonal and local time variation of the quiet-time LSTIDs is similar to that of the medium-scale traveling ionospheric disturbances (MSTIDs) propagating equatorward (e.g., [37,58,59]). Both of them occur frequently during the daytime in winter. The equatorward traveling MSTIDs are also suggested to be induced by AWGs. We speculate that the background wind field has a significant influence on both the LSTIDs and MSTIDs, and so there is a similarity between them when they are traveling in the same direction.

## 5. Conclusions

Aside from the GPS network, the dense OI ionosonde network offers another possibility to continuously observe the horizontal propagation of TIDs by both day and night. It is an economical solution using multi-input and multi-output technology. The inversion parameters of the network can present the propagation characteristics of the oscillations on a horizontal plane through the peak plasma density variations at each observation point. Based on the observations of the OI ionosonde network in North China and the digisondes in Beijing, Mohe and Yakutsk, a statistical study of the quiet-time LSTIDs was conducted. An interesting phenomenon of the mid-latitude LSTIDs of quasi-3-h period has been reported. It highlights the observation particularity of the OI ionosonde network, and the long-term observations of this network will deepen our understanding of the atmosphere–ionosphere coupling as well as the influence of momentum from polar regions on the middle and low latitudes.

## Figures and Tables

**Table 1:** The occurrence number and occurrence rate of the quiet-time LSTIDs in four seasons from 1 September 2009 to 21 August 2011.

Season	Autumn	Winter	Spring	Summer
Number of events	29	110	16	2
Occurrence rate	27.0%	56.7%	16.3%	1.0%

**Figure A1:**
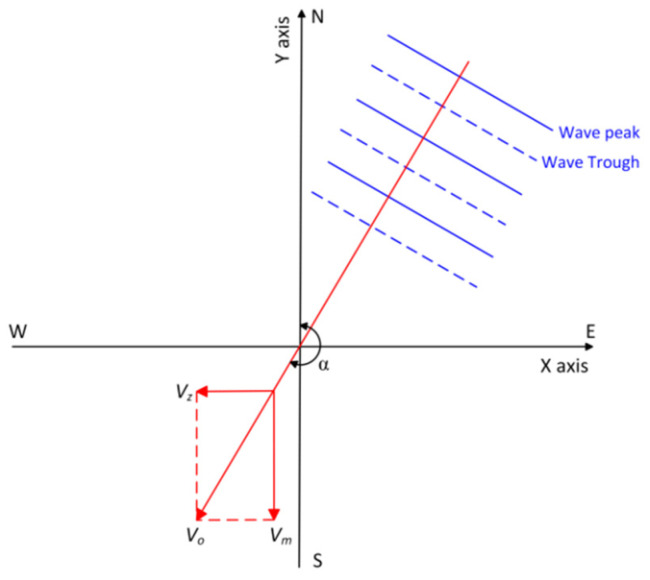
Schematic diagram of the propagating parameters of the periodic disturbances in the horizontal plane. The azimuth α is clockwise from north. The letters N, E, S, and W are used to indicate the directions of north, east, south and west, respectively.

## Data Availability

The Beijing and Mohe digisonde data can be obtained from the Chinese Meridian Project (http://159.226.22.74/ (accessed on 28 December 2021)). The data of the oblique-incidence ionosonde network can be obtained from the China seismo-ionospheric ground-based monitoring network (http://www.sign.ac.cn/ (accessed on 8 April 2021)). The data of the Yakutsk digisonde can be obtained from the Lowell GIRO Data Center (http://giro.uml.edu/ (accessed on 28 December 2021)). The Kp indexes can be obtained from the World Data Center for Geomagnetism Web interface (http://wdc.kugi.kyoto-u.ac.jp (accessed on 28 December 2021)).

## Appendix A

Computing method for the wave speed and azimuth.

Given a periodic disturbance traveling with the speed *V_o_* and azimuth *α* as indicated in Figure A1, the zonal velocity *V_z_* and meridian velocity *V_m_* of the disturbance are

*V_z_* = *V_o_* sin *α* (A1)

*V_m_* = *V_o_* cos *α* (A2)

The projection speeds of the disturbance on the horizontal and vertical axes are *V_x_* and *V_y_*. It must be pointed out that a disturbance is not a point target, and thus *V_y_* ≠ *V_m_* and *V_x_* ≠ *V_z_*. *V_x_*/*V_y_* can be estimated by the slope of the wavefront in the longitude/latitude–time plot. When *V_x_* and *V_y_* are known, the wave speed *V_o_* and wave azimuth *α* can be calculated as follows:

(A3) $$V_{x}=V_{z}+V_{m}\cot\alpha =V_{z}+\frac{V_{m}^{2}}{V_{z}}=\frac{V_{z}^{2}+V_{m}^{2}}{V_{z}}=\frac{V_{o}^{2}}{V_{z}}=\frac{V_{o}}{\sin\alpha }$$

(A4) $$V_{y}=V_{m}+V_{z}\tan\alpha =V_{m}+\frac{V_{z}^{2}}{V_{m}}=\frac{V_{m}^{2}+V_{z}^{2}}{V_{m}}=\frac{V_{o}^{2}}{V_{m}}=\frac{V_{o}}{\cos\alpha }$$

(A5) $$\tan\alpha =\frac{V_{z}}{V_{m}}$$

By the Equations (A3) and (A4), we know

(A6) $$V_{x}\sin\alpha =V_{y}\cos\alpha \Rightarrow \tan\alpha =\frac{V_{y}}{V_{x}}$$

So,

(A7) $$\alpha =\arctan\frac{V_{y}}{V_{x}}$$

(A8) $$V_{o}=V_{y}\cos\alpha =V_{x}\sin\alpha$$

Given that the slope of the wave peak and wave trough in the latitude–time and longitude–time plots are *l_m_* and *l_z_* (°/s), respectively:

(A9) $$V_{y}=l_{m}R_{o}$$

(A10) $$V_{x}=l_{z}R_{o}\cos37{}^{\circ}$$

where *R_o_* represents the distance of 1° latitude (or 1° longitude at the equator) on the Earth’s surface and the mean latitude of the ionosonde network is 37°. So, the wave azimuth *α* and the wave horizontal speed *V_o_* can be also expressed as

(A11) $$\alpha =\arctan\left(\frac{l_{m}}{l_{z}\cos37{}^{\circ}}\right)$$

(A12) $$V_{o}=l_{m}R_{o}\cos\alpha$$
